# Sustainable concrete: investigating the synergistic effects of coconut fiber, wheat straw ash, and silica fume on RAC strength and durability

**DOI:** 10.1038/s41598-025-02234-1

**Published:** 2025-07-08

**Authors:** Ahmed A. Alawi Al-Naghi, Tariq Ali, Inamullah Inam, Muhammad Zeeshan Qureshi, Nabil Ben Kahla, Nejib Ghazouani, Hawreen Ahmed

**Affiliations:** 1 Civil Engineering Department, University of Ha’il, 55476 Ha’il, Kingdom of Saudi Arabia; 2 Department of Civil Engineering, Swedish College of Engineering and Technology, Wah, 47080 Pakistan; 3 Department of Civil Engineering, Engineering Faculty, Laghman University, Mehtarlam, Afghanistan; 4 Department of Civil Engineering, University of Engineering and Technology, Taxila, Pakistan; 5 Department of Civil Engineering, College of Engineering, King Khalid University, PO Box 394, 61411 Abha, Kingdom of Saudi Arabia; 6 Center for Engineering and Technology Innovations, King Khalid University, 61421 Abha, Kingdom of Saudi Arabia; 7 Department of Civil Engineering, College of Engineering, Northern Border University, 73222 Arar, Kingdom of Saudi Arabia; 8 Mining Research Center, Northern Border University, 73222 Arar, Kingdom of Saudi Arabia; 9 Department of Highway and Bridge Engineering, Technical Engineering College, Erbil Polytechnic University, Erbil, 44001 Iraq

**Keywords:** Coconut fiber, Wheat straw ash, Recycled aggregate, Mechanical and durability properties, Engineering, Materials science

## Abstract

As concrete production accounts for a large percentage of worldwide CO_2_ emissions, there is a need for alternative sustainable construction materials. Simultaneously, the increasing generation of construction and demolition waste has led to the growing interest in using recycled aggregates (RA) in concrete. However, recycling aggregate (RAC) tends to demonstrate poor mechanical and durability performance because of relatively high porosity and weak interfacial transition zone existing in RA. This study explores the synergistic effects of coconut fiber (CF), wheat straw ash (WSA) and silica fume (SF) in its enhancement of RAC performance. Mechanical (compressive and tensile strengths) and durability (water absorption and acid resistance) characteristics of RA (50%, 75%, and 100%) incorporated with various proportions of WSA (5%, 10%, and 15%) have been studied. Additionally, CF (1.5%) and SF (7%) were also added in all mixtures. The findings show that the optimum mix (10% WSA and 50% RA) achieves a compressive strength of 30.7 MPa at 90 days. The tensile strength was also improved, with the 10% WSA mix offering the highest tensile strength of 3.89 MPa at 90 days. Durability tests showed that water absorption decreased, and acid resistance improved with the addition of WSA, especially with 10% WSA, which had the lowest water absorption of 4.8%. Microstructural Analysis of the concrete matrix showed, particularly for mixes with increased WSA content, indicate lower porosity and better bonding. The present work establishes base evidence for the use of CF, WSA and SF in improving the performance and sustainability of RAC and is a viable option for construction applications, particularly in the presence of the high construction and demolition waste regions.

## Introduction

Concrete is a prevalent construction material globally^1,2^, which experiences substantial environmental costs mostly due to the high energy requirements and greenhouse gas emissions associated with cement manufacturing^3–6^. The yearly concrete production globally, according to current estimates, is approximately 1600 million metric tons, which produces 7–10% carbon dioxide into the environment^7^. As a result of these environmental issues, there are now worldwide concerns with building material prices, raw material shortages, energy use, and the utilization of solid waste as a substitute^8^. At the same time, the increase in the production of C&W (construction and demolition) waste has heightened interest in the use of recycled aggregates (RA) as a potential new source of aggregate for concrete^9^. Recycled aggregate concrete (RAC) generally possesses inferior mechanical and durability properties because RA have high porosity and weak interfacial transition zones^10^. It has also been observed that the mechanical and durability characteristics of concrete made using recycled aggregates are greatly influenced by the properties of the parent concrete such as mix proportions and workability^11^. Consequently, concrete with recycled aggregates tends to exhibit lower mechanical properties than that made with natural limestone or granite aggregates primarily due to higher porosity and poor bond interface between new and old concrete^12^. However, mineral admixtures and pozzolanic materials can enhance these characteristics, as C–S–H gel fills the porous microstructure of RA, thus improving the mechanical performance and durability of the materials.

Similarly to the partial substitution of cement with supplementary cementitious materials (SCMs)^13^, the utilization of recycled aggregates decreases the demand for natural aggregates^14^, hence mitigating the adverse environmental effects of building. Recycled aggregates usually consist of 35%–20% of attached old cement binder paste and 65–80% of natural aggregates^15^. The impact of silica fume (SF) in structural concrete has been the focus of numerous studies^16–18^.The incorporation of silica fume in concrete enhances mechanical characteristics, abrasion resistance, permeability and drying shrinkage^18–22^. Furthermore, additional binders can greatly increase the strength and durability of concrete such as rice husks or straws ash^23,24^ millet husks^25^, Peanut and corn cobs ash^26^, sugar cane bagasse^27,28^, palm shale oil^29^. Wheat straw ash (WSA) has recently been considered as a promising material for the applications as SCM. It is made by the calcination of Wheat Straw, a waste material from agriculture industry, it has high SiO_2_ content and exhibits pozzolanic behavior. Using WSA can effectively decrease the overall CO_2_ emissions by decreasing the clinker content in concrete, increase the quality of concretes^30–35^. The production of wheat globally is approximately 750 million tons from 2016 to 2017. Pakistan is one of the largest providers of wheat globally, the annually wheat production of Pakistan is estimated to be 26.6 million tons^36,37^.

coconut fiber (CF) is extensively studied for its capacity to enhance concrete performance by improving energy absorption, flexibility, and minimizing microcrack formation^38–40^. It was determined that a CF measuring 5 cm in length and containing 2% exhibited better mechanical characteristics compared to PC. The improved toughness of NF (natural fibers) was the rationale for the selection of CF in prior research^41–43^. The incorporation of SCMs in combination of fibers is recognized to improve the mechanical and durability characteristics of cement-based materials. Although many studies have been developed to evaluate the benefits of mineral admixtures, SCMs, and fibers applied singly to RAC^10,11,44–49^, few studies have been conducted on the use of CF and WSA as a partial cement replacement for strength and durability of concrete. In order to minimize cement content in concrete, recycle building waste, and improve material qualities, additional materials and fiber as reinforcements have been explored.

Efforts to reduce cement use, recycle construction waste, and enhance material properties have driven the exploration of supplementary materials and fiber reinforcements. Among the various combinations investigated recently, the incorporation of recycled aggregates (RA), coconut fiber (CF), wheat straw ash (WSA), and silica fume has not been examined, perhaps representing the optimal combination for the development of high-performance and sustainable concrete. To develop a comprehensive sustainable concrete design strategy, it is critical to understand and characterize the synergistic interactions of these materials. In this study a detailed experimentation is conducted and the mechanical properties, durability performance, and microstructural characteristics of fiber-reinforced RAC incorporating WSA and silica fumes as dual SCMs are investigated. Optimizing these materials in terms of their proportions and interactions aims to ground for a scientific basis to advance RAC as a sustainable and high-performance construction material.

## Materials and methods

**Fig. 1:**
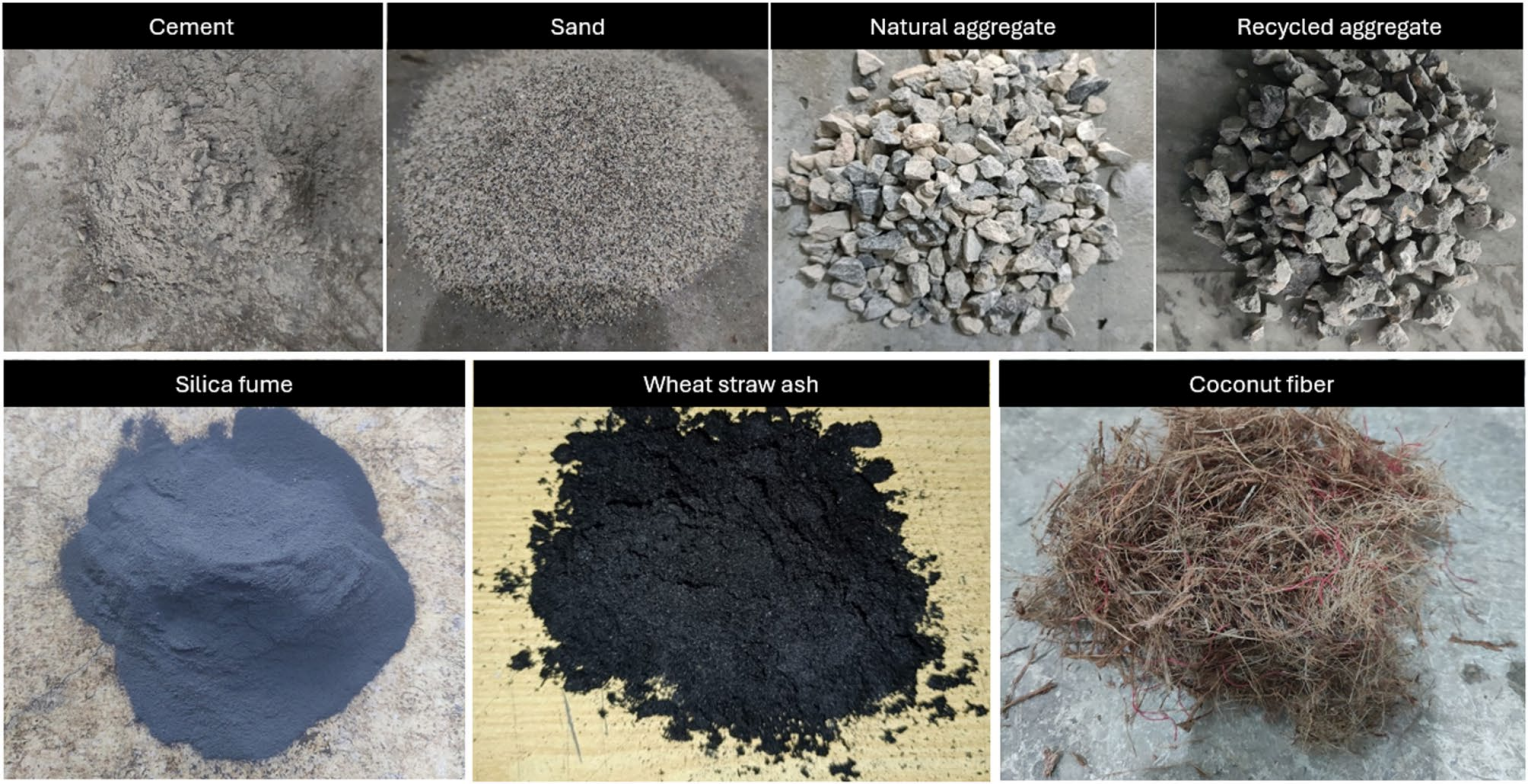
Materials used in this study.

The disposal of building demolition waste presents a significant challenge. In this study RCA (Recycled coarse aggregate) has been utilized as a substitute for normal aggregate. Despite this, it has been noted that the compressive, tensile, and bonding strengths of concrete decreased when RCA was utilized independently as opposed to regular aggregate. The absorption of water is the primary issue with recycled aggregate concrete due to the presence of attached mortar. The addition of SCMs within RAC can mitigate this challenge. This research employs supplementary cementitious materials (SCMs), specifically wheat straw ash (WSA) and silica fume (SF), in combination with coconut fiber (CF), to enhance the mechanical and durability characteristics of Recycled aggregate concrete. There is a lack of research on the use of recycled coarse aggregate with rice straw ash, silica fume and coconut fiber. There has been some research on the effects of using wheat straw ash in cement on its own. Whenever compared to other SCMs and fiber, the environmental and economic benefits of using wheat straw ash are superior. The materials are shown in Fig. 1.

This study includes the combination of Recycled Coarse Aggregate at proportions of 50%, 75%, and 100% with wheat straw ash at levels of 5%, 10%, and 15%, while maintaining a consistent addition of silica fume at 7% and coconut fiber at 1.5%. Type I cement in accordance with ASTM C150^56^ were employed. WSA and SF can be utilized as pozzolanic material in accordance with ASTM C311^57^. CF are used according to ASTM C1116. The fiber is separated from the shell and cut it into a length of 50 mm. Locally available fine aggregate (fineness modules = 2.65) and coarse aggregate are used according to ASTM C136 and ASTM C127 respectively. The chemical properties of OPC, WSA and SF are shown in Table 1 while the physical properties of OPC and coconut fiber are displayed in Table 2. Table 3 presented the physical properties of NCA and RCA.

**Table 1:** Chemical composition of cement, wheat straw ash and silica fume.

Composition	OPC (%)	WSA (%)	SF (%)
Al_2_O_3_	4.35	22.89	0.1–0.5
SiO_2_	21.38	49.10	80–95
CaO	61.58	11.78	0.1–1
MgO	3.10	2.46	0.2–1.5
Fe_2_O_3_	2.29	5.30	0.1–0.8
Na_2_O	1.15	0.08	0.1–0.9
K_2_O	1.74	3.15	0.3–1.2
SO_3_	1.99	0.75	0.1–0.3
LOI	2.42	4.49	< 6

**Table 2:** Physical properties of OPC and CF.

Cement		Coconut fiber	
Parameters	Properties	Parameters	Properties
Fineness	7	Bulk density	1.15–1.45 g/cm^3^
Soundness	3 mm	Diameter	0.1–0.5 mm
Residue (insoluble)	0.25%	Tensile strength	100-200 MPa
Consistency	26.5%	Water absorption	130–180%
Initial setting time	35 min	Moisture content	8–12%
Final setting time	405 min	–	–

**Table 3:** Physical properties of NCA and RA.

Properties	NCA	RCA
Specific gravity	2.81	2.75
Fineness Modulus	4.40	5.11
Bulk density	1572 kg/m^3^	1485 kg/m^3^
Absorption	2.80%	4.75%

### Selection and rationale of a variable

The variables are the key factors in the design of concrete mixes which ensure the optimization of mechanical performance and sustainability. The synergistic impact of selected silica fume (7%), coconut fiber (1.5%), and wheat straw ash (5%, 10%, and 15%) focuses on further enhancing the performance of Recycled Aggregate Concrete (50, 75 and 100%). The inclusion of silica fume is attributed to its pozzolanic activities that increase the concrete’s strength and lower its permeability by additional C–S–H gel formation, thus improving the bond between aggregates and the cement matrix at the interface. Coconut Fiber was added to improve the tensile strength and toughness because the fibers serve to reduce microcracking and improve the load transfer between particles which tends to mitigate the brittleness of RAC. The use of wheat straw ash was preferred due to its favorable environmental aspects and pozzolanic action which improves the durability of the concrete by minimizing water absorption and increasing the resistance to acid attack while also occupying the pores within the matrix. These materials together mitigate the critical concerns of RCA such as poor interfacial transition zones (ITZ), excessive porosity, weakening mechanical properties, and improve the strength, durability, and sustainability of RAC for structural use^11,50–53^.

Previous studies have demonstrated that WSA can improve compressive strength (at optimal replacement levels of 10–15%), reducing permeability, and improving long term durability. The composition of cement-based composites depends on the type of fiber^54,55^ and the fiber length for better adhesion to the matrix^55^, treatment of the fibers^56^, and the correct dispersion of the fiber in the matrix^57,58^. For mechanical requirements of composites, tensile, flexural, and impact strengths are the most studied tests in several other studies^56,59–61^. However, adding of 0.5–1.5% coconut fiber has increased the compressive strength of reference concrete by about 20–25%^38^.

### Preparation and mix design of specimens

A total of 336 cylinders of size 150 × 300 mm were prepared in this study. The compressive strength and tensile were performed according to ASTM C39 and ASTM C496 after 7, 28 and 90 days of curing period. The acid resistance was performed according to ASTM C267 using 5% H_2_SO_4_ Solution at 1 month and 3 months period. The water absorption was performed in accordance with ASTM C642. The mix design was conducted in accordance with ACI 211.1. The specimen was named WSA-RCA (wheat straw ash -recycled concrete aggregate) with different percentages of WSA and RCA. The mixed proportions are shown in Table 4.

**Table 4:** Mix proportion of the samples.

Mix ID	OPC (kg/m^3^)	WSA %age	SF (kg/m^3^)	CF FIBER (kg/m^3^)	FA (kg/m^3^)	RCA %age	RCA kg/m3	NCA %age	NCA (kg/m^3^)	WSA kg/m^3^	W/C
WSA0-RCA0	350	0	25	5.25	655	0	0	100	1055	0	0.55
WSA0-RCA50	350	0	25	5.25	655	50	527.5	50	527.5	0	0.55
WSA0-RCA75	350	0	25	5.25	655	75	791.25	25	263.75	0	0.55
WSA0-RCA100	350	0	25	5.25	655	100	1055	0	0	0	0.55
WSA5-RCA0	332.5	5	25	5.25	655	0	0	100	1055	17.5	0.55
WSA5-RCA50	332.5	5	25	5.25	655	50	527.5	50	527.5	17.5	0.55
WSA5-RCA75	332.5	5	25	5.25	655	75	791.25	25	263.75	17.5	0.55
WSA5-RCA100	332.5	5	25	5.25	655	100	1055	0	0	17.5	0.55
WSA10-RCA0	315	10	25	5.25	655	0	0	100	1055	35	0.55
WSA10-RCA50	315	10	25	5.25	655	50	527.5	50	527.5	35	0.55
WSA10-RCA75	315	10	25	5.25	655	75	791.25	25	263.75	35	0.55
WSA10-RCA100	315	10	25	5.25	655	100	1055	0	0	35	0.55
WSA15-RCA0	297.5	15	25	5.25	655	0	0	100	1055	52.5	0.55
WSA15-RCA50	297.5	15	25	5.25	655	50	527.5	50	527.5	52.5	0.55
WSA15-RCA75	297.5	15	25	5.25	655	75	791.25	25	263.75	52.5	0.55
WSA15-RCA100	297.5	15	25	5.25	655	100	1055	0	0	52.5	0.55

Moreover, the use of CF, WSA, SF, and RAC in mix design must adhere to the specifications laid out by several design codes, as they are fundamental in defining the performance standards for construction purposes. Although CF is not specifically covered under most design codes, its use is covered under the recommendations for fiber-reinforced concrete laid out in standards including ACI 318 and BS 8500. WSA, as a supplementary cementitious material (SCM), complies with ASTM C618 and BS 1377 standards, which outlines the guidelines for pozzolanic materials, usually suggesting a replacement of cement between 5 and 15%. SF improves strength and durability of concrete regulated by ASTM C618, BS 1377 up to 10% substitution According to ACI 555R, up to 100% of Recycled Coarse Aggregate (RCA) can be used in concrete, as long as it complies with relevant performance and durability standards for aggregates in concrete, as outlined in EN 206 (European Standard for Concrete). The present study investigates the efficacy of using 100% replacement of RCA and deals intensively with the improvement of concrete durability via incorporation of RCA together with various supplementary materials CF (1.5%), WSA (5%, 10%, and 15%) and SF (7%) in order to evaluate its performance towards achieving high performance concrete with improved durability. These findings adhere to these standards and therefore validate the sustainable application of RCA in construction works.

## Results and discussion

### Compressive strength

**Fig. 2:**
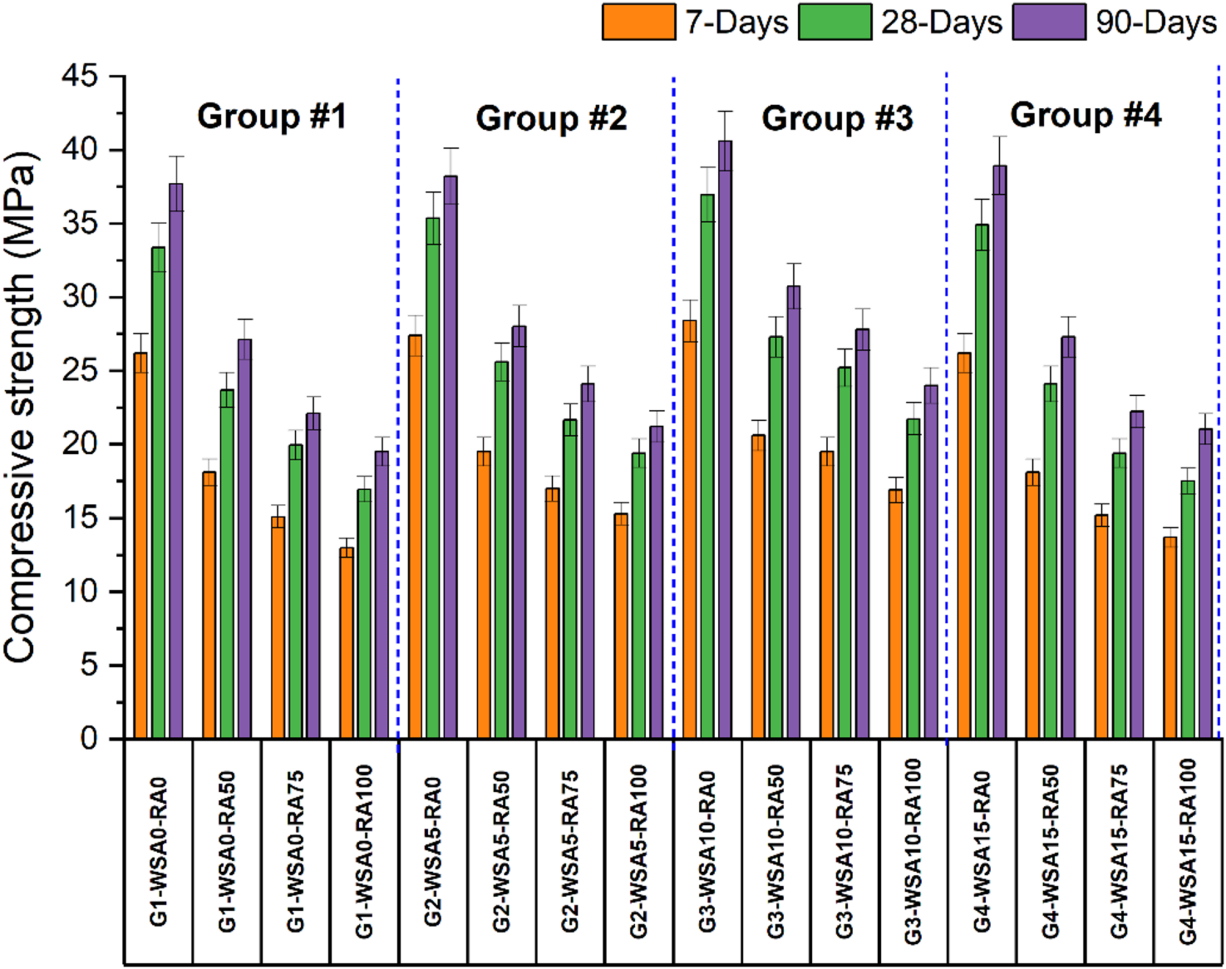
Compressive strength of different groups at 7, 28 and 90 days.

Figure 2 shows the effect of RA and WSA on concrete compressive strength at each curing age. Recycled aggregates (RA) were employed to replace coarse aggregates at four levels: 0%, 50%, 75%, and 100%. This research evaluates the impact of wheat straw ash addition on concrete composed of silica fume and coconut fiber, categorizing all concrete mixtures into four primary groups with 0%, 5%, 10%, and 15% wheat straw ash. The strength of concrete declines with RA cooperation at various replacement levels (50%, 75%, and 100%), regardless of the WSA percentage. Results indicate that increasing content of wheat straw ash (WSA) usually improves compressive strength especially at later curing ages. In Group 1 (without WSA), the strength is found to be 33.4 MPa. This decreases to 23.7 MPa with 50% RCA and further drops to 20 MPa with 75% RCA. The lowest strength, 17 MPa, is recorded with 100% RCA. In Group 2 (with 5% WSA), the strength of control is found to be 35 MPa. Which decreases to 25.6 MPa, 21.7 MPa and 19.4 MPa with the addition of 50%, 75% and 100% RCA respectively. The maximum increase in strength is found with the addition of 10% WSA, the results reveal that in Group 3 (with 10% WSA), the control strength is found to be 37 MPa which decreases to 27.3 MPa with 50% RCA, and further drops to 25 MPa with 75% RCA and 21.7 MPa with 100% RCA. While in Group 4 (with 15% WSA) it was found to reveal better results than Group-1 but lower strength than Group-3 as per the results statistics the strength of control is found to be 34 MPa. Which decreases to 24.1 MPa, 19.4 MPa and 17.5 MPa with the addition of 50%, 75% and 100% RCA respectively.

The mix (G3-WSA10-RA0), which was the best performing Group-3 mix, reached 40.6 MPa strength at 90 days, which is significantly higher than mixes that do not contain recycled aggregate (RA). As RA content increases, the strength decreases, with the lowest strength (19.5 MPa at 90 days) seen with G1-WSA0-RA100 (100% RA). For the 50% RA (e.g. G2-WSA5-RA50 and G3-WSA10-RA50), the results drop in strength but show significant improvement over higher RA content. Pozzolanic properties of WSA help to increase strength development, and this effect is particularly pronounced at later curing ages^30^. The interfacial transition zone (ITZ) in RCA concrete is weaker than that of conventional concrete. This weakness arises because the RCA contains adhered mortar from the original concrete. The compromised ITZ can lead to micro-cracking, which reduces the overall strength^62^. Load transfer in RCA concrete happens through the ITZ or at direct contact points between aggregates. There are three main types of ITZs in RCA concrete: one between the aggregates and the new mortar, another between the old mortar and the new mortar, and a third within the RCA itself, where the aggregate meets the old, adhered mortar^63,64^.The pozzolanic properties and filling effect of WSA enhanced the compressive strength of the mixture when the substitution level of WSA reached 10%^30^. However, when the concentration of WSA and the quantity of cement developed, the required CH (calcium hydroxide) for reacting with silica reduced leading to a reduction in strength. This can be attributed to the fibers’ ability to facilitate aggregate penetration into the pores for optimal bonding; however, an increase in fiber volume leads to bulkiness, distorting the bond and diminishing strength^30^.

The increase in strength of concrete with the incorporation of WSA can be attributed to the pozzolanic reaction that occurred due to the presence of amorphous silica^32^. The results confirm that the initial strength is not affected by the incorporation of WSA but the pozzolanic reaction becomes more active with passage of time, contributing to the later strength of the specimen. The maximum increase in strength is recorded for Group-3 (having 10% WSA) beyond this certain point, the strength begins to decline, primarily because the amount of primary cementitious material is reduced.

### Tensile strength

**Fig. 3:**
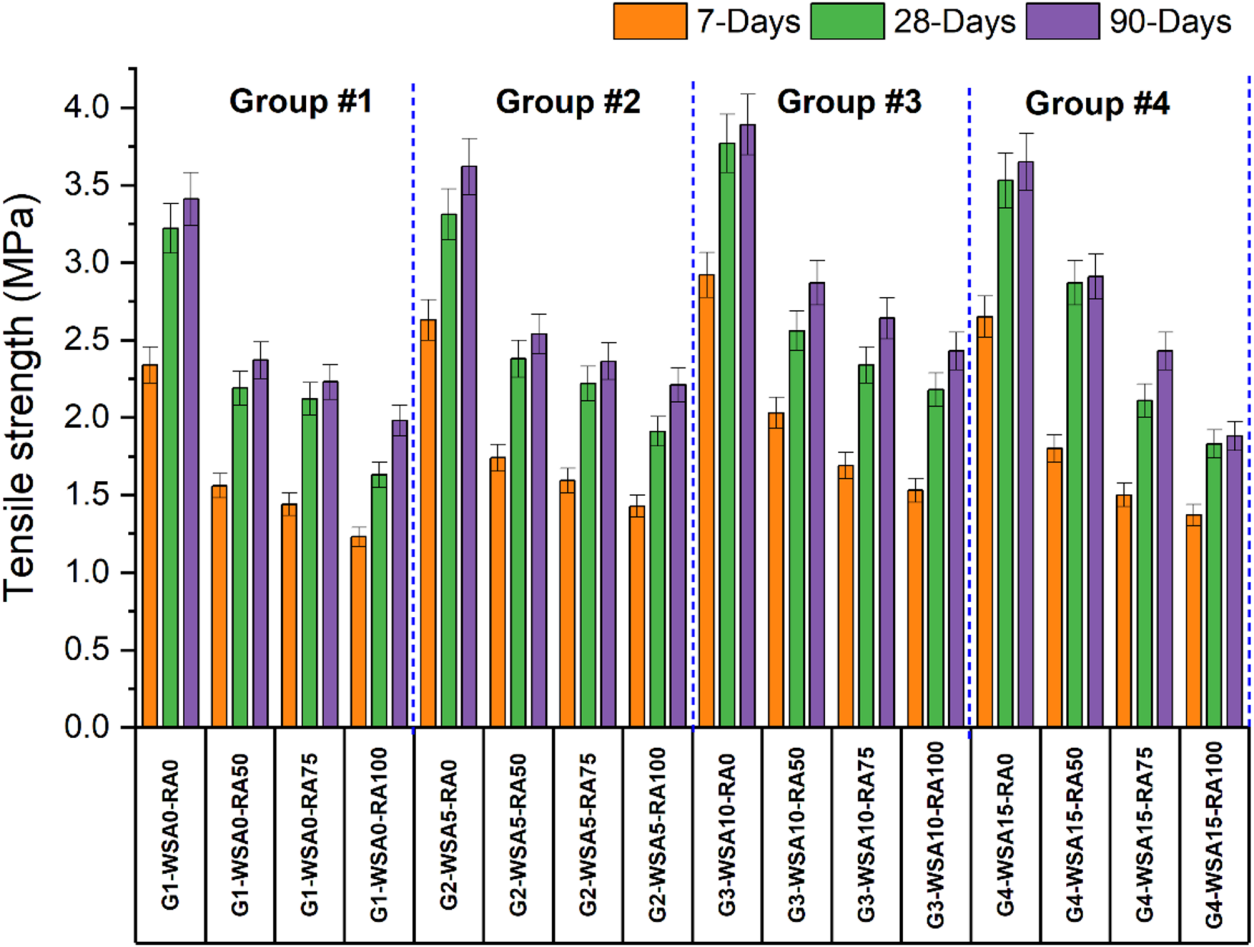
Tensile strength of different groups at 7, 28 and 90 days.

Figure 3 illustrates the development of tensile strength in concrete mixtures with different replacement ratios of natural coarse aggregate (NCA) by recycled concrete aggregate (RCA), with variable quantities of wheat straw ash (WSA) at different curing ages. The findings suggest that adding WSA generally enhances the tensile strength, especially at later curing stages. The result analysis of 7-day strength confirms that in Group 1 (without WSA), the control mixture achieved a tensile strength of 2.34 MPa, which decreased to 1.56 MPa with 50% RCA, further dropping to 1.44 MPa with 75% RCA. The lowest tensile strength, 1.23 MPa, was recorded with 100% RCA. In Group 2 (with 5% WSA), the control tensile strength was 2.63 MPa, which decreased to 1.74 MPa, 1.59 MPa, and 1.43 MPa with the incorporation of 50%, 75%, and 100% RCA, respectively. The best results were observed with the addition of 10% WSA. In Group 3 (with 10% WSA), the control tensile strength reached 2.92 MPa, and while it decreased to 2.03 MPa with 50% RCA, further reductions to 1.69 MPa and 1.53 MPa were observed with 75% and 100% RCA, respectively. Group 4 (with 15% WSA) showed better results than Group 1 but lower tensile strength than Group 3. In Group 4, the tensile control strength was 2.65 MPa, and it decreased to 1.80 MPa, 1.51 MPa, and 1.37 MPa with the addition of 50%, 75%, and 100% RCA, respectively.

Furthermore, the result analysis of 28-day strength confirms that in Group 1 (without WSA), the control mixture achieved a tensile strength of 3.22 MPa, which decreased to 2.19 MPa with 50% RCA, further dropping to 2.12 MPa with 75% RCA. The lowest tensile strength, 1.63 MPa, was recorded with 100% RCA. In Group 2 (with 5% WSA), the control tensile strength was 3.31 MPa, which decreased to 2.38 MPa, 2.22 MPa, and 1.91 MPa with the incorporation of 50%, 75%, and 100% RCA, respectively. The best results were observed with the addition of 10% WSA. In Group 3 (with 10% WSA), the control tensile strength reached 3.77 MPa, and while it decreased to 2.56 MPa with 50% RCA, further reductions to 2.34 MPa and 2.18 MPa were observed with 75% and 100% RCA, respectively. Group 4 (with 15% WSA) showed better results than Group 1 but lower tensile strength than Group 3. In Group 4, the tensile control strength was 3.53 MPa, and it decreased to 2.87 MPa, 2.11 MPa, and 1.83 MPa with the addition of 50%, 75%, and 100% RCA, respectively.

Similarly, the result analysis of 90-day strength confirms that in Group 1 (without WSA), the control mixture achieved a tensile strength of 3.41 MPa, which decreased to 2.37 MPa with 50% RCA, further dropping to 2.23 MPa with 75% RCA. The lowest tensile strength, 1.98 MPa, was recorded with 100% RCA. In Group 2 (with 5% WSA), the control tensile strength was 3.62 MPa, which decreased to 2.54 MPa, 2.36 MPa, and 2.21 MPa with the incorporation of 50%, 75%, and 100% RCA, respectively. The best results were observed with the addition of 10% WSA. In Group 3 (with 10% WSA), the control tensile strength reached 3.89 MPa, and while it decreased to 2.87 MPa with 50% RCA, further reductions to 2.64 MPa and 2.43 MPa were observed with 75% and 100% RCA, respectively. Group 4 (with 15% WSA) showed better results than Group 1 but lower tensile strength than Group 3. In Group 4, the tensile control strength was 3.65 MPa, and it decreased to 2.91 MPa, 2.43 MPa, and 1.88 MPa with the addition of 50%, 75%, and 100% RCA, respectively.

The main reason for the reduction of tensile strength in recycled concrete aggregate (RCA) concrete is the low strength of the transition zones between the aggregates and the mortar^64,65^. Residual mortar on recycled aggregates causes microcracks leading to stress concentration and crack propagation and weakening of the material^66^. In conventional concrete, the load transfer in the strongest sense occurs at the aggregate-to-aggregate interface, while the weakest link is typically the aggregate to mortar contact, or interfacial transition zone (ITZ), which is relatively more porous and less dense^67^. However, the load transfer in RCA concrete is structurally more complex than in ordinary concrete, as it involves weaker contact points, such as between recycled aggregate and old mortar, as well as between old mortar particles^68,69^.

Strength of concrete was significantly increased with the incorporation of Waste Straw Ash into concrete, especially in splitting tensile strength. This is consistent with previous research, showing that WSA is a good way to increase concrete strength and to promote the use of industrial waste^30,32,70,71^.

### Water absorption

**Fig. 4:**
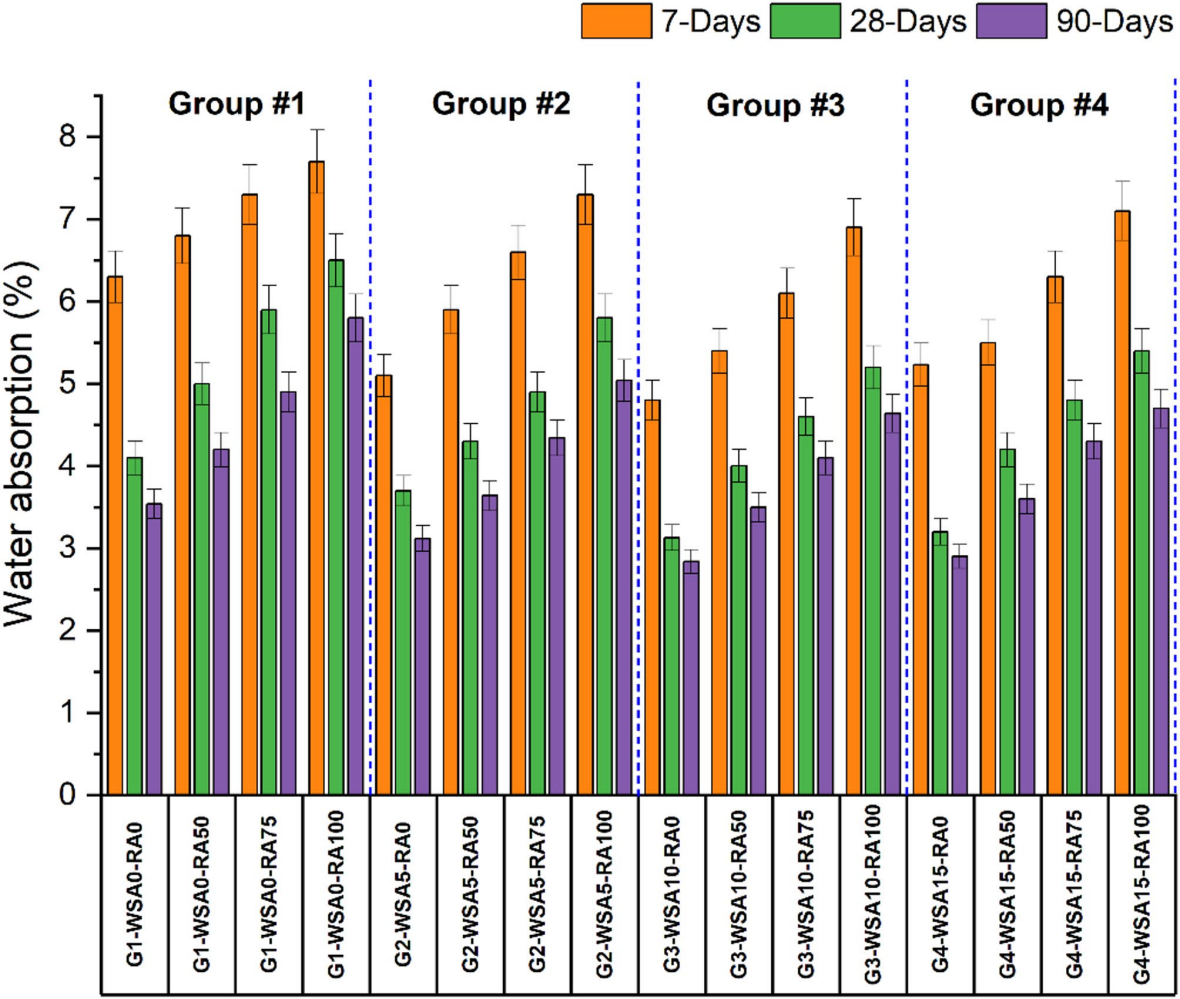
Water absorption of different groups at 7, 28 and 90 days.

The water absorption test on the concrete specimens is carried out and the results of incorporating wheat straw ash (WSA) and Recycled Concrete Aggregate (RCA) on concrete specimens are shown in Fig. 4. It ensures the pores existence in the microstructure and addresses the durability aspect of concrete. RA incorporation at different replacement levels (50%, 75% and 100%) the water absorption (WA) of concrete is found to be increasing irrespective of WSA percentage. The results indicate that increasing content of wheat straw ash (WSA) usually improves impermeability especially at later curing ages. In Group 1 (without WSA), the water absorption (WA) is found to be 6.3%. This increases to 6.8% with 50% RCA, and further increased to 7.3% with 75% RCA. The highest WA 7.7% is recorded with 100% RCA. In Group 2 (with 5% WSA), the water absorption (WA) is found to be 5.1%. Which increases to 5.9%, 6.6%, and 7.3% with the addition of 50%, 75% and 100% RCA respectively. The maximum decrease in water absorption (WA) is found with the addition of 10% WSA, the results reveal that in Group 3 (with 10% WSA), the water absorption is found to be 4.8% which increases to 5.4% with 50% RCA, and further increased to 6.1% with 75% RCA and 6.9% with 100% RCA. While in Group 4 (with 15% WSA) it was found that its impermeability in terms of water absorption is better than Group-1 but lower than Group-3 as per the results statistics, water absorption (WA) of control is found to be 5.2%. Which increases to 5.5%, 6.3% and 7.1% with the addition of 50%, 75% and 100% RCA respectively.

RCA has higher porosity than natural aggregates because it contains adhered mortar and microcracks resulting from the recycling process^65,72^. This porous nature of RCA creates additional voids within the concrete matrix, and this makes the concrete vulnerable to take in more water, leading to the concrete’s long term durability degradation^72^. RCA-based concrete is also commonly more permeable, which makes it more vulnerable to chemical attack and water-related deterioration^73^. It has also been demonstrated that the addition of wheat straw ash (WSA) improves the water resistance of concrete. As a pozzolanic material, WSA reacts with calcium hydroxide (produced during cement hydration) to form additional cementitious compounds acting as a binder, filling the pores with no increase in the overall porosity of the concrete. The reduction in porosity results in decrease in water absorption and increased durability of the concrete^74^. Using WSA and RCA together however, the improvements achieved by WSA in water absorption may at least partially offset the negative water absorption effects contributed by RCA. The use of WSA at optimized dosage may overcome the increase in water absorption due to RCA, as WSA has a pozzolanic effect^36^. Thus, by using these two individual techniques synergistically as components of evaluation, overall performance of concrete mixes with respect to water absorption should be balanced, this being more sustainable and durable. These results are like previous research which suggests that supplementary concrete materials such as WSA reduce the detrimental effects of RCA on the concrete durability in the presence of water saturation^75^. The corresponding figure illustrates the results of the water absorption test for concrete mixes with various percentages of WSA and RCA.

WSA particles, as being finer than cement particles, enhance concrete density and reduce its porosity via different mechanisms. First, their fine size is reinforcing at the scale between voids and cement particles, creating a denser matrix. The concrete’s impermeability as well as its durability are enhanced by this "filler effect," reducing the capillary porosity. Second, the hydration of WSA causes additional calcium silicate hydrate (C–S–H) gel to form, and which densifies and improves the strength of the concrete. Additionally, the finer WSA particles also help in better hydration in the early stages, with better formation of C–S–H gel and better microstructure. This leads to less microcracking and better ability to resist environmental degradation (e.g. water absorption and chemical attacks). This means that, in terms of WSA incorporation, we get stronger, more durable concrete which is also less permeable^76^.

### Acid attack

**Fig. 5:**
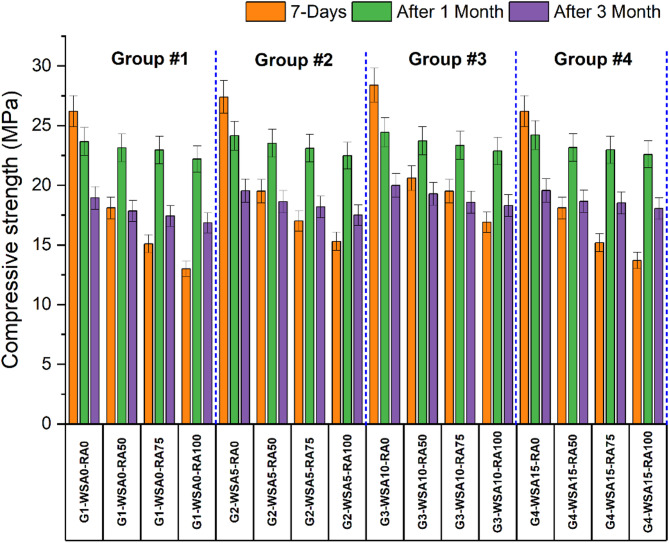
Reduction in compressive strength of different groups after 1 and 3 months.

To evaluate the durability and the performance of different mixes in an acid environment the test of acid curing for concrete specimens is carried out comprised of different amounts of recycled aggregates along with the optimum dosages of supplementary materials such as WSA and coconut fiber (CF) and compare it with conventional concrete mixes. The specimens are tested according to standard guidelines, in particular ASTM C267, while the 5% sulfuric acid concentration is used for all mixes to compare their performance in acidic exposure. The specimens are then immersed and evaluated for weight loss and strength degradation. The results depicted as shown in Fig. 5 represent the loss in strength recorded after 1 month and 3 months for the different concrete mixes which provides a relative way of comparing the performance of the different concrete mixes.

Some of the samples were tested and others are kept for curing in an acid environment for a further 1-month and 3-month period to evaluate the comparative performance of different groups. It is found that for 0%RCA the strength is 26.2 Mpa in Group-1 (with no WSA) while the strength with 50%, 75%, 100% of RCA percentage were found to be 18.1 Mpa, 15.2 Mpa and 13 Mpa respectively. Strength reduction is observed in these mixes after being kept in an acid environment for 1 month and strength reduced by 9.7%, 11.7%, 12.4% and 15.3% respectively. The test results also confirm that the mixes suffered further strength loss when mixes were kept further for 3 months periods and for this period 27.7%, 31.9%, 33.5% and 35.7% strength reduction is observed respectively.

Similarly, the strength of Group-2 (with 5% WSA) is 27.4 Mpa for 0% RCA, while the strength was 19.5 Mpa, 17 Mpa and 15.3 Mpa for 50%, 75% and 100% of RCA respectively. Which reduced 7.8%, 10.2%, 11.8% and 14.2% respectively after 1-month acidic exposure and after 3-months the reduction is 25.45%, 28.9%, 30.6% and 33.2% respectively in same environment.

Similarly For Group-3 (with 10% WSA) is 28.4 Mpa for 0% RCA, while the strength was 20.60 Mpa, 19.5 Mpa and 16.9 Mpa for 50%, 75% and 100% of RCA respectively. Which reduced 6.8%, 9.5%, 10.9% and 12.7% respectively after 1-month acidic exposure and after 3-months the reduction is 23.7%, 26.4%, 29% and 30.1% respectively in same environment.

Furthermore, for Group-4 (with 15% WSA) is 26.2 Mpa for 0% RCA, while the strength was 18.1 Mpa, 15.3 Mpa and 13.7 Mpa for 50%, 75% and 100% of RCA respectively. Which reduced 7.6%, 11.6%, 12.3% and 13.8% respectively after 1-month acidic exposure and after 3-months the reduction is 25.2%, 28.8%, 29.4% and 31.3% respectively in same environment.

Several reasons exist as to why the presence of Recycled Concrete Aggregate (RCA) particles in concrete can result in its lower performance in acidic environments^65,77^. RCA usually contains old mortars that are more porous than new concrete. The increased in porosity allows sulfuric acid to penetrate a larger surface area, accelerating degradation. The reaction of adhered mortar in RCA is chemically different from the new matrix and it can react more aggressively with sulfuric acid to form calcium sulfate (gypsum) and weaken the structure. In addition, ITZs between recycled aggregate and new mortar in RCA concrete tend to be weaker, so more prone to microcracking. The accumulation of these microcracks then allow acid to penetrate deeper into the material, exacerbating the degradation process even more^77,78^. In addition, the mechanical recycling process inherently leads to internal microcracks in RCA particles themselves, which serve as ways for acid penetrate and accelerate damage. In addition, RCA concrete often has lower cementitious content than normal concrete, which further weakens it against acid attack. The lower durability and reduced density of RCA also make it more porous, thereby providing additional avenues for the acid to attack and degrade the concrete. Thus, RCA concrete suffers higher weight loss and lower strength in acidic environments when compared to normal concrete mixes^79,80^.

## Microstructural analysis

**Fig. 6:**
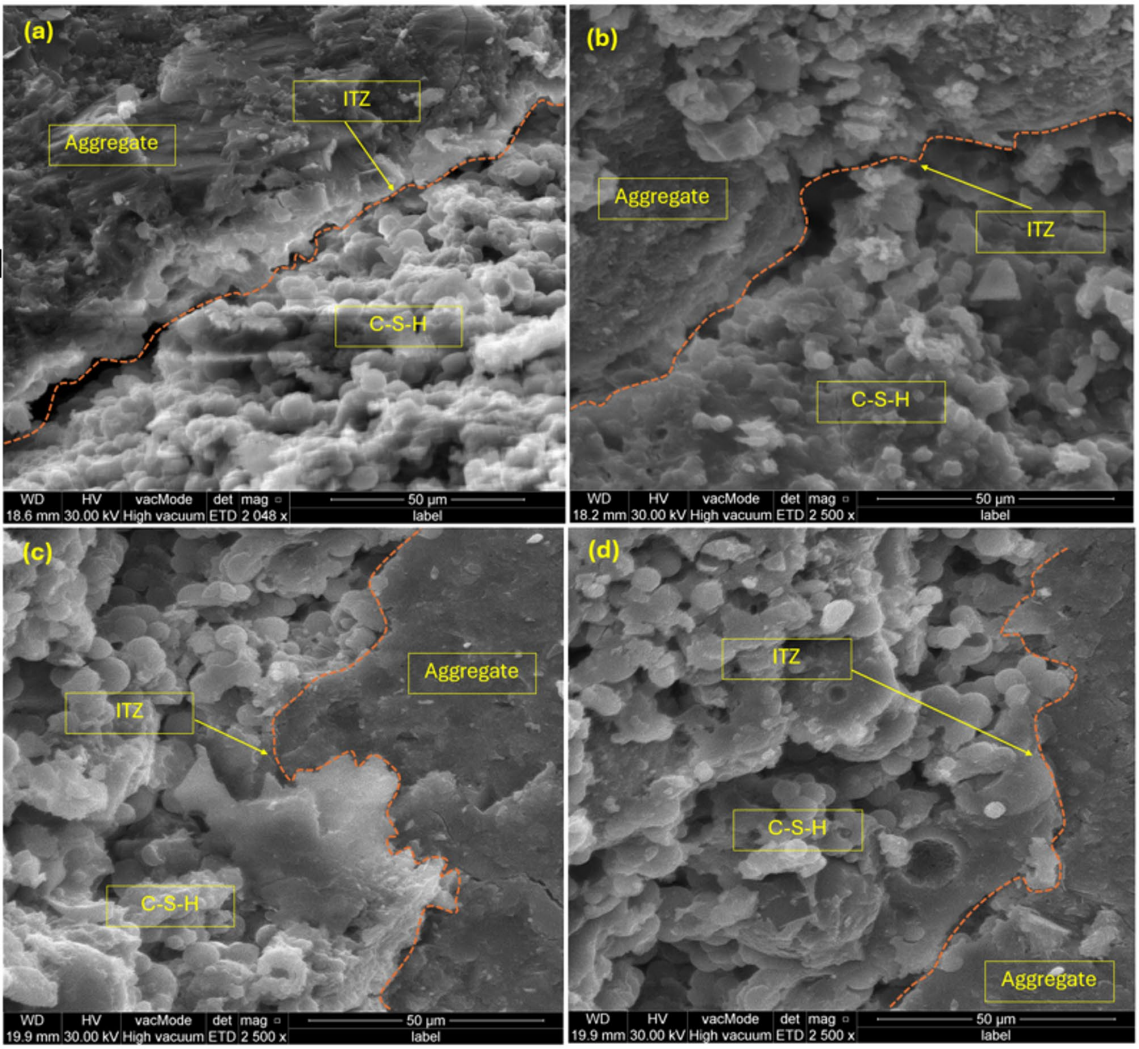
SEM analysis (**a**) WSA0-RA100, (**b**) WSA5-RA100, (**c**) WSA10-RA100, (**d**) WSA15-RA100.

The factors affecting the service life and durability of concrete structures are determined basically by the flow properties of concrete. In particular, the pore structure of the hydrated cement paste is directly coupled to these flow characteristics. The impact of adding wheat straw ash (WSA) at different replacement levels (0%, 5%, 10%, 15%) with 100% RA on concrete’s microstructure is analyzed using scanning electron microscopy, as shown in Fig. 6. Concrete is a viscoelastic material, where flow properties are key to its durability and service life, and these properties are influenced by the pore structure of the hydrated cement paste. Supplementary materials such as wheat straw ash (WSA) can greatly affect the microstructure of Concrete and enhance its performance. WSA, a byproduct of agricultural waste, is viewed as a potential alternative as a supplementary cementitious material based on its pozzolanic properties. WSA modifies the microstructure of concrete not only as a filler but also as a pozzolanic agent resulting in denser concrete with enhanced mechanical properties and durability. In the case of concrete containing WSA, the SEM images show large plate crystals present within the material, which indicate that the material has gaps or voids within the matrix, and dark patches that confirm the presence of calcium hydroxide (CH) crystals. Often the result of incomplete hydration, these voids can poison the concrete’s mechanical strength and its durability. Still, the CH crystals also indicate ongoing hydration, from distribution and from size they can suggest details of the hydration process. WSA addition increases the density of the concrete microstructure by filling the voids and reacting with calcium hydroxide to further harden the calcium silicate hydrate (C–S–H) gel, which provides an additional densification agent for the concrete microstructure. This reaction is important for increasing bonding within the cement matrix and reducing porosity. The effectiveness of WSA incorporation into concrete is dependent on the WSA particle size. WSA particles having a finer size tend to have greater surface area resulting in higher reactivity and more significant pozzolanic effects. Small particles fill the voids in the concrete better, creating a more compact, well balanced concrete structure. Typically, SEM images of concrete with finer WSA particles reveal denser C–S–H gel with less porosity, indicating finer particles contribute to a stronger bond between the cement particles, reducing overall void space. This results in better strength and durability because a denser microstructure is less likely to be damaged by environmental factors such as water ingress, freeze–thaw cycles, and chemical attacks. On the other hand, the microstructure obtained by employing coarser WSA particles is less compact. For coarse WSA particle cement samples, larger voids, and less formation of C–S–H gel, result in lower mechanical strength, and reduced durability. However, the coarser, less reactive less parts do not participate as effectively in the pozzolanic reaction, decreasing C–S–H gel formation and increasing porosity. In aggressive environments where high durability is necessary, this can undermine the structural integrity of concrete. Both facings and the pozzolanic action of WSA improve the microstructure as well as overall strength and durability under service conditions. When reacting with calcium hydroxide from cement hydration, WSA forms additional C–S–H gel and strengthens the concrete while reducing microcrack formation. In a study^81^, it showed that pozzolanic action results in C–S–H formation, reduced micro cracks formation and more durable products. The improved microstructure improves the resistance of the concrete to various environmental challenges, for instance, chemical attacks or freeze thaw cycles. The increased microstructure and performance of concrete induced by the WSA particles further supports that finer WSA particles are more effective than coarser WSA for improving concrete performance. It is a more durable, high strength material resulting from a combination of the filler effect, which reduces voids and improves packing density, and the pozzolanic effect, which strengthens the bond and forms additional C–S–H gel. This means that besides allowing the environmental impact of the production of concrete to be reduced, the use of WSA as a supplemental cementitious concrete material enhances production of stronger and more durable concrete structures.

## Performance and economic analysis

Cost analysis shows that various mixes tested reveals significant insights into the performance and economic efficiency of WSA10 with recycled aggregate. Group 3, specifically the WSA10RCA0 mix, demonstrated the highest compressive strength, achieving 40.6 MPa at 90 days, while also offering the most economical performance with the highest EI index (strength/rate ratio) of 0.000925. This indicates that WSA10RCA0 provides the best balance between strength and cost. However, as recycled aggregate was added to the mix, a gradual reduction in compressive strength was observed. Notably, the WSA10R50 mix, which incorporates 50% recycled aggregate, achieved a compressive strength of 30.7 Mpa, well within the acceptable range, and had a commendable EI index of 0.000829. This makes WSA10R50 a promising choice for applications involving WSA and recycled aggregates in cement-based structures, as it maintains a good balance of strength and cost-effectiveness, making it suitable for both structural and commercial applications. Table 5 displays the cost of each material per kg while Table 6 shows the cost analysis of each mix.

**Table 5:** Price list of each material.

Material	Price/Kg (Pakistani rupee)
OPC	33
WSA	10
SF	150
CF	4
NFA	11
RCA	8
NCA	21

**Table 6:** Cost analysis with respect to EI index.

Mix ID	Rate/kg (PKR)	WSA0-RCA0	WSA0-RCA50	WSA0-RCA75	WSA0-RCA100	WSA5-RCA0	WSA5-RCA50	WSA5-RCA75	WSA5-RCA100	WSA10-RCA0	WSA10-RCA50	WSA10-RCA75	WSA10-RCA100	WSA15-RCA0	WSA15-RCA50	WSA15-RCA75	WSA15-RCA100
OPC	33	11,550	11,550	11,550	11,550	10,972.5	10,972.5	10,972.5	10,972.5	10,395	10,395	10,395	10,395	9817.5	9817.5	9817.5	9817.5
SF	150	3750	3750	3750	3750	3750	3750	3750	3750	3750	3750	3750	3750	3750	3750	3750	3750
CF fiber	4	21	21	21	21	21	21	21	21	21	21	21	21	21	21	21	21
FA	11	7205	7205	7205	7205	7205	7205	7205	7205	7205	7205	7205	7205	7205	7205	7205	7205
RCA	8	0	4220	6330	8440	0	4220	6330	8440	0	4220	6330	8440	0	4220	6330	8440
NCA	21	22,155	11,077.5	5538.75	0	22,155	11,077.5	5538.75	0	22,155	11,077.5	5538.75	0	22,155	11,077.5	5538.75	0
WSA	10	0	0	0	0	175	175	175	175	350	350	350	350	525	525	525	525
Water	0	0	0	0	0	0	0	0	0	0	0	0	0	0	0	0	0
Total Rate		44,681	37,823.5	34,394.8	30,966	44,278.5	37,421	33,992.3	30,563.5	43,876	37,018.5	33,589.8	30,161	43,473.5	36,616	33,187.3	29,758.5
CS (90 days)		37.7	27.1	22.1	19.5	38.2	28	24.1	21.2	40.6	30.7	27.8	24	38.9	27.3	22.2	21
EI		0.000844	0.000716	0.000643	0.000630	0.000863	0.000748	0.000709	0.000694	0.000925	0.000829	0.000828	0.000796	0.000895	0.000746	0.000669	0.000706

## Conclusion

- The utilization of WSA (5%, 10%, and 15%) into the concrete enhances the strength of the control samples by 6%, 10.8%, and 4.6%, respectively. However, for concrete with 100% Recycled Coarse Aggregate (RCA), strength gain was 8.2%, 15%, and 3.5% at these same WSA replacement levels, illustrating the positive impact of WSA on concrete strength even when using recycled aggregates.
- The use of wheat straw ash (WSA) in concrete improves its durability, especially its acid resistance and water absorption. These results demonstrate that the incorporation of WSA (5%, 10%, and 15%) has a significant influence on water absorption, with maximum efficiency recorded at 10% WSA dosage. The concrete mix with 10% WSA yielded a 4.8% water absorption rate which is notably lower than the control mix’s 6.3%. For acid resistance, 10% WSA also claimed best results achieving the lowest strength loss percentage of 6.8% after 1 month and 23.7% after 3 months acid exposure.
- The optimum value of WSA for structural concrete is discovered to be 10% that enhances strength of RCA concrete significantly as 25 Mpa strength is achieved even with 75% RCA, however based on the entire performance and results it is recommended that combination of 50% RCA with 10% WSA can be used for all purposes of structural applications.
- The cost analysis of the tested mixes shows that the WSA10RCA0 mix, with no recycled aggregate, is the best trade-off between strength and costs making the 40.6 MPa at 90 days and the EI index of 0.000925. Compressive strength decreases as recycled aggregate is added, but the WSA10R50 mix with 50% recycled aggregate still provides an acceptable 30.7 MPa and an EI index of 0.000829. The good performance with recycled materials and low costs offered by WSA10R50 makes it a cost effective and strong choice for structural and commercial applications.

In conclusion this research determines that the incorporation of wheat straw ash (WSA) into Recycled Coarse Aggregate Concrete (RAC) increases strength and durability. The combined use of CF fibers and two types of SCMs (WSA and silica fume) does not only improve the mechanical properties but also improve the ITZ which contributes to better load transfer and durability. The combination of the given materials offers a scientific basis for enhancing high performance and sustainability of RCA in construction. Controlling concrete strength can be improved by 6%, 10.8% and 4.6% with the addition of WSA (5%, 10% and 15%), and 100% RCA concrete by 8.2%, 15%, and 3.5%. WSA enables greater durability with reduced water absorption and increased acid resistance yielding a denser, less permeable microstructure. Optimal WSA content for structural concrete is 10%, leading to significant strength increases as tested even with 75% RCA. The cost analysis shows that WSA10RCA0 exhibits the best strength to cost ratio, while WSA10R50, which has 50% recycled aggregate, found to be an economical and good balanced sustainable strength mix (30.7 MPa). For cost effective high performance structural concrete, this study recommends the use of 10% WSA with 50% RCA.

## Data Availability

The data is available from the corresponding author upon request.
